# Metagenomic Profiling of Known and Unknown Microbes with MicrobeGPS

**DOI:** 10.1371/journal.pone.0117711

**Published:** 2015-02-02

**Authors:** Martin S. Lindner, Bernhard Y. Renard

**Affiliations:** 1 Research Group Bioinformatics (NG4), Robert Koch Institute, Berlin, Germany; Wilfrid Laurier University, CANADA

## Abstract

Microbial community profiling identifies and quantifies organisms in metagenomic sequencing data using either reference based or unsupervised approaches. However, current reference based profiling methods only report the presence and abundance of single reference genomes that are available in databases. Since only a small fraction of environmental genomes is represented in genomic databases, these approaches entail the risk of false identifications and often suggest a higher precision than justified by the data. Therefore, we developed MicrobeGPS, a novel metagenomic profiling approach that overcomes these limitations. MicrobeGPS is the first method that identifies microbiota in the sample and estimates their genomic distances to known reference genomes. With this strategy, MicrobeGPS identifies organisms down to the strain level and highlights possibly inaccurate identifications when the correct reference genome is missing. We demonstrate on three metagenomic datasets with different origin that our approach successfully avoids misleading interpretation of results and additionally provides more accurate results than current profiling methods. Our results indicate that MicrobeGPS can enable reference based taxonomic profiling of complex and less characterized microbial communities. MicrobeGPS is open source and available from https://sourceforge.net/projects/microbegps/ as source code and binary distribution for Windows and Linux operating systems.

## Introduction

Recent advances in experimental and computational technologies have increased the number and diversity of sequenced microbial organisms. Today, single cell sequencing [1, 2] and metagenome assembly [3] allow extracting the genomic sequences even for uncultivable bacteria. With the increasing number of microbial reference sequences, reference based metagenomic analysis methods became significantly more powerful and popular [4–7].

Although the taxonomic resolution of reference based methods in whole genome sequencing metagenomic experiments is higher than for other strategies such as 16S rRNA [8] or composition based taxonomic profiling [9], these methods encounter a different problem: the reference genome databases are still far from complete and—due to constant evolution—will never be. In practice, the often proposed species or strain level accuracy [5] is only achieved if sufficient sequenced strains of the organism of interest are available. Otherwise, these methods are at risk of suggesting accuracy to the user that is not justified by the underlying reference data when they report the presence of a species in the database which happens to be the closest sequenced relative to the organism in the sample. For example, the NCBI bacterial genomes contain the *Akkermansia muciniphila* ATCC BAA-835 genome, which is the only representative of the class *Verrucomicrobiae* in the database. If a related *Verrucomicrobium* is sequenced, current tools are likely to report *A. muciniphila* ATCC BAA-835 without warning the user that the identified strain has considerable difference to the true organism. This holds both for abundance estimation and for read binning approaches.

This problem becomes evident when considering current approaches for assessing the composition of microbial communities via metagenomics. For example, MetaPhlAn [4] is a fast and popular metagenomic abundance estimation tool which maps reads to a set of selected marker sequences that are unique for each organism in the database. The marker sequences are carefully selected such that a read can only match to one marker and can therefore be assigned to a distinct organism. Therefore, the abundances of organisms can be easily calculated by extrapolating the number of reads on the marker sequences to the whole genome. Together with the small size of the marker sequence database, this makes MetaPhlAn very fast while the accuracy is comparable to other reference based methods. However, since the genomes are reduced to short marker sequences, there is no possibility to detect or even quantify differences between the sequenced organism and the reference. Organisms that contain marker sequences of different reference strains (e.g. by horizontal gene transfer) show up in the results multiple times and it is not possible to detect such cases. A second approach based on marker genes is mOTUs [10], which is inspired by profiling methods using the 16S rRNA phylogenetic marker gene. Here, the authors selected a set of ten marker genes that are more discriminative for phylogenetic inference and do not suffer from differing copy numbers. Using whole genome sequencing data instead of amplicon sequencing, the reads are mapped to the marker genes and the taxonomic profile of the sample is calculated. Since the selected marker sequences are single copy genes, mOTUs also allows species abundance estimation. However, due to the marker based approach, mOTUs suffers from the same drawbacks as MetaPhlAn. Another recent approach for both abundance estimation and read binning is Pathoscope [5], a method that analyzes read alignments to whole genomes with particular focus on reads mapping to multiple genomes. The program calculates a probability for each read alignment that is used in a Bayesian mixture model. The reads are then reassigned to their most likely origin by finding the optimal model parameters. This allows Pathoscope to differentiate between highly similar strains even for very low sequencing depth. Although Pathoscope is able to identify the closest related reference when the true genome is not present in the database, it is not clear from the results whether the reported organism is a perfect match or not. There is a variety of further tools available, however, to the best of our knowledge, none of the existing tools quantifies the distance between reference genome and organism in the dataset.

Here, we present MicrobeGPS, a tool that accurately identifies microbial organisms in metagenomic sequencing data, estimates their abundance, and quantifies their distances to known reference genomes. In contrast to current methods which typically estimate the abundances of available reference genomes, MicrobeGPS approaches the problem from a biological perspective and finds microbial organisms that are then described with suitable reference genomes. Here, a microbial organism is characterized by its sequencing depth in a similar fashion as the composition-based AbundanceBin [11] method. MicrobeGPS searches for reads that are likely to originate from genomic regions that are unique to one organism in the dataset. Based on this information, MicrobeGPS creates clusters of reference genomes supporting the same candidate organism. The supporting genomes determine the taxonomic affiliation of the candidate while the genome–dataset validity score [12] quantifies the distance between candidate and supporting genomes. This observation driven approach makes MicrobeGPS unique in the sense that the tool reports highly accurate identifications and abundances in the case of suitable reference genomes. Otherwise, it describes the contained organism using the closest related known reference genomes providing a sound quantification of sequence disagreement. The graphical user interface eases data analysis and interpretation as it provides interactively browsable results, color representation of quantitative traits, as well as interactive graphs and taxonomy trees.

## Methods

**Fig 1:**
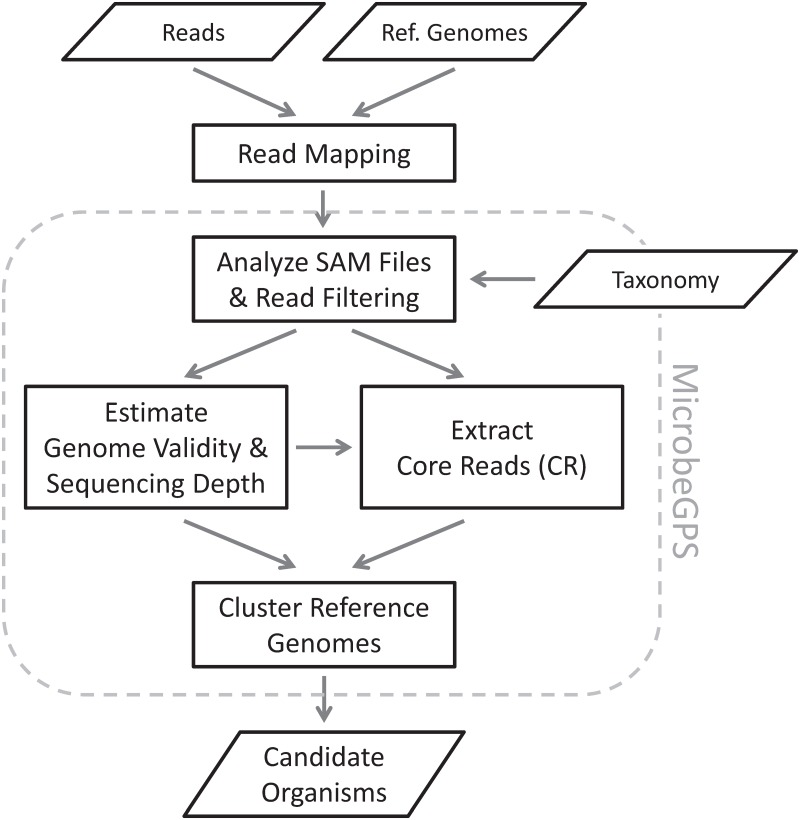
MicrobeGPS workflow. As a first step, MicrobeGPS reads and analyzes the SAM files of the metagenomic reads mapped to a set of reference genomes. Early filtering of the reads helps reducing the amount of data by discarding reads that are not meaningful for MicrobeGPS. Then, MicrobeGPS estimates the Genome Dataset Validity score and the local sequencing depth of each reference genome and uses this information to extract *core reads* (CR), reads that are presumably unique for a particular organism in the sample. Based on the CR and the shared reads, MicrobeGPS clusters the reference genomes into groups, where each group represents a single biological candidate organism in the sample.

**Fig 2:**
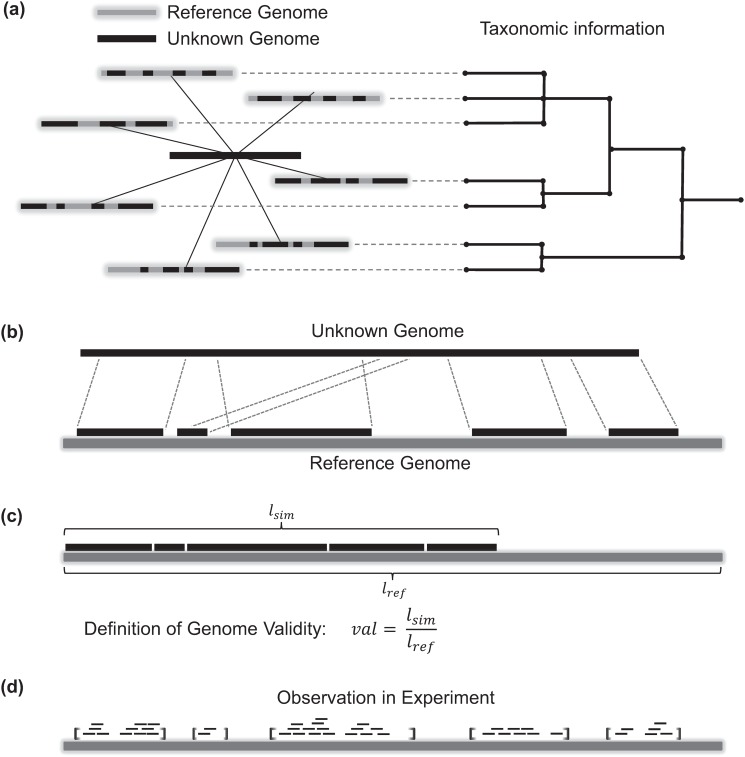
GPS principle and GDV score. (a) The taxonomic *location* of an unknown organism (dark) is estimated by calculating distances to already known and taxonomically classified reference genomes (light), inspired by the global positioning system GPS. The GDV measures the distance between references and organism: (b) The available reference genome shares parts of its sequence with the unknown genome in the sample. (c) The GDV of the reference genome with respect to the unknown genome is defined as the fraction of the reference genome that agrees with the unknown genome. (d) Since the unknown genome is realized as a set of short reads obtained by sequencing a biological sample, the GDV of the reference genome with respect to the unknown genome can be estimated from the sequencing reads mapped to the reference genome as described in [12].

The MicrobeGPS method is outlined in Fig. 1 and is described in this section. The fundamental idea behind MicrobeGPS is that we use already characterized reference genomes to *describe* the identity of the (possibly unknown) organisms in a metagenomic dataset (see Fig. 2a). This approach contrasts current methods that typically seek to directly identify and quantify reference genomes (species/strains) or higher taxa in the dataset. In analogy to the global positioning system GPS, MicrobeGPS estimates the taxonomic position of an organism in the sample by measuring the genomic distance between the organism and the closest related reference genomes. In contrast to taxonomic binning approaches (such as Pathoscope or Megan [13]), MicrobeGPS does not assign each read to a single taxon, but operates on the whole dataset to identify the taxa present in the sample.

The distance between reference genome and the organism is measured by the genome–dataset validity (GDV) as follows. Consider an unknown organism in the sample that shares parts of its genome sequence with a known genome (Fig. 2b). Then, the GDV is defined as the fraction of the reference genome that has a counterpart in the unknown genome as shown in Fig. 2c. Since the unknown genome is realized as a set of short reads, the true GDV of the reference genome is not directly observable. In practice, not all shared parts of the reference genome may be covered by reads (Fig. 2d). Therefore, the GDV is estimated as described below based on prior work in [12].

MicrobeGPS builds on the assignment of reads to a collection of reference genomes. Based on coverage depth observed locally on the reference genomes, MicrobeGPS identifies reads that are likely to originate from a genomic region that is unique to one of the organisms in the sample. These reads are then used together with the other shared reads to create clusters of reference genomes, where each cluster represents a single organism in the dataset. The following sections describe the details of our method.

### Local sequencing depth and GDV estimation

A metagenomic dataset contains sequencing reads derived from an environmental community of microbial organisms. In a whole genome NGS experiment, the number of reads from each organism can be assumed to be proportional to its genome length and its abundance in the community. The reads are sampled from random positions in the genome such that we assume a homogeneous sequencing depth on the original genomes. Apart from influences such as extreme GC bias, this assumption is justified for the current NGS devices [14].

As a first step, MicrobeGPS requires the sequencing reads to be mapped to a broad collection of microbial reference genomes. Considering the vast amounts of data produced by current sequencers, it makes sense to use a fast read mapper that reports all or the N best read mappings. We had good experience with Bowtie 2 [15], NGM [16] and Masai [17], but other tools supporting the SAM format as output are also feasible.

MicrobeGPS excludes reads from the analysis that originate from highly conserved regions, such as 16S rRNA reads. Since these reads can be mapped to almost all bacterial genomes they are not informative in our setup and can suppress the faint signals of very low abundant organisms. Furthermore, MicrobeGPS discards reference genomes without sufficient supporting reads or a highly uneven read distribution on the genome. Multiple reference sequences (chromosomes, plasmids) belonging to the same organism are grouped at this stage.

In the next step, we estimate the sequencing depth in the covered regions on each reference genome. For that purpose we compose a mixture distribution of three probability distributions:

$$\begin{array}{c} f\left(x|\bar{\alpha },\lambda \right)=\alpha_{1}\cdot z\left(x\right)+\alpha_{2}\cdot P\left(x|\lambda \right)+\alpha_{3}\cdot T_{\lambda }\left(x\right). \end{array}$$

*z* is the zero distribution as used in [18] to construct a zero-inflated Poisson distribution in combination with the Poisson distribution *P. T*_*λ*_ is a Poisson tail distribution as introduced in [12] to account for skews in the Poisson distribution due to differences between reference genome and sequencing reads. The *α*_*i*_ are the mixing coefficients. This mixture distribution is fitted to the histogram of genome coverage depths of each reference genome using a modified EM algorithm, providing estimates for the parameter *λ* and the mixing coefficients *α*_*i*_. Then, the parameter *λ* of the Poisson distribution represents the sequencing depth on the genomic regions shared between reference genome and the organism in the sample.

The genome–dataset validity score (GDV) is defined as the fraction of the reference genome for which there is evidence in the dataset. Therefore, the GDV can be calculated from the mixing coefficient of the zero distribution: GDV = 1 − *α*_1_. As shown in the original publication, the zero-inflated Poisson distribution with tail provides more accurate estimates of the GDV for sequencing depths ≥ 1× than the negative binomial counterpart. A modification of this approach to ultra–low sequencing depths (≤ 1×) is described in detail in S1 Text. In brief, the histogram of genome coverage depths is replaced by the histogram of distances between read start positions and the mixture distribution consists of three geometric distributions. In practice, the former approach is used for sequencing depths ≥ 1×, the latter for sequencing depths < 1×. With this strategy, MicrobeGPS is able to recover the GDV and the original sequencing depth of an organism down to 0.05× even if the closest related available reference genome has only low similarity.

The GDV takes values between 0 (reference genome has no similarity to an organism in the sample) and 1 (reference genome is completely represented in the sample). In practice, GDV scores larger than 0.8 almost always indicate highly relevant reference sequences. Scores below 0.2 should always be treated with caution, as they may either indicate a very distant relation or can be the result of spurious read mappings. In a metagenome where the majority of constituents are unknown, GDV scores higher than 0.2 can be typically regarded as informative, although it should be clear that a species level identification is not appropriate.

### Reference genome clustering

Once the local sequencing depths are estimated, MicrobeGPS extracts all core reads (CR) from the dataset. The CR are a subset of all reads {*r*} and are defined as

$$\begin{array}{c} CR=\left\{r\;|\;\frac{\max\left(D_{r}\right)-\min\left(D_{r}\right)}{\text{mean}(D_{r})}\leq t\right\}, \end{array}$$

where D_*r*_ are the sequencing depths of reference genomes that the read *r* maps to. In other words, a read *r* is a CR, when it maps to multiple reference genomes with similar local sequencing depth, i.e. the maximum relative difference between the sequencing depths on the target genomes of a read does not exceed the user defined limit *t* (default: *t* = 0.2). Generally, a higher *t* leads to more reads classified as CR because larger differences in sequencing depth are allowed. This can be useful for datasets with novel organisms or with low sequencing depth, where the local sequencing depth estimation may be not as accurate as for high sequencing depth datasets. CR are likely to originate from a genomic region that is unique to one particular organism with respect to all other organisms in the sample: if the read would originate from a non-unique genomic region and could be assigned to multiple organisms, it is likely that these organisms have different sequencing depths. However, it is still possible that two or more organisms have the same sequencing depth such that false CR are identified; these cases require specialized treatment as described in the following steps.

At this stage, MicrobeGPS still works on the level of reference genomes, where it is not clear how the number of reference genomes relates to the number of true organisms. While a very uncommon bacterium might be represented by only a single reference genome, the presence of a well-studied model organism in the sample, such as *Escherichia coli*, will make a lot more reference genomes appear meaningful. Therefore, MicrobeGPS creates clusters of reference genomes in the final step, where each cluster represents a single organism in the dataset. First, MicrobeGPS clusters reference genomes sharing high numbers of CR using a greedy strategy: a reference genome is compared to all existing clusters and the fraction of shared CR out of all shared reads are computed. If the reference genome shares sufficient CR with the existing clusters, MicrobeGPS assigns the genome to the cluster with the highest overlap. Requiring a minimum CR overlap with clusters (default: 20% of all CR) allows accounting for organisms with similar sequencing depth: the number of CR shared with a cluster representing a different organism is typically lower than the total number of CR. If a lower CR overlap is used, the method requires less CR to be shared between reference genomes in order to assign them to the same cluster. This, however, bares the risk of falsely assigning genomes to the same cluster that represent different organisms. On the other hand, requiring a high overlap may lead to multiple clusters representing the same organism. If no suitable cluster can be identified based on the CR, MicrobeGPS searches in a second step for clusters with a very high fraction of shared reads (default: 60% of all reads are shared between reference genome and cluster). The influence of this parameter is similar to the minimum CR overlap. However, we set a higher default value here, as the fraction of shared reads is not as discriminative as the fraction of shared CR. Finally, a new cluster is created if the genome cannot be assigned to any existing cluster based on the shared CR or all shared reads. This greedy clustering strategy has two advantages over existing clustering schemes: First, the number of clusters is not required beforehand as for k-means [19]. Second, it is not necessary to calculate a full distance matrix between the reference genomes as for hierarchical clustering [20], which is computationally expensive. Furthermore, clustering can be sped up if scientific names or genome identifiers are available for the reference sequences. Then, taxonomically related genomes are clustered previous to other genomes, reducing the number of necessary comparisons. Compared to other traditional clustering approaches, such as hierarchical clustering, our approach was designed with focus on low run time rather than mathematical exactness and proves to be robust in application.

The clustering step has quadratic complexity in the number of reference genomes for the worst case when each reference genome is used to create a new cluster. However, with reasonable choice of parameters (e.g., default), the complexity is between linear and quadratic in practice, depending on the structure of the reference genome collection and the composition of the microbial community. With the number of reads *M* and the number of reference genomes *N*, the worst case complexity of MicrobeGPS is *O*(*MN* ^2^). However, due to the greedy algorithm, the average case complexity is *O*(*MN*) in practice.

In summary, each cluster is built up of one or more reference genomes and represents one organism in the sample, identified by its sequencing depth and the reads shared between the supporting genomes. If taxonomic information is provided with the reference genomes, MicrobeGPS assigns the lowest common ancestor taxon name of all supporting genomes to the candidate to facilitate interpretation of the results. Discrimination between organisms with similar sequencing depth is achieved by requiring a minimum overlap of reference genomes for clustering such that only highly similar organisms with equal sequencing depth are at risk to be falsely regarded as one organism. The distances between the identified candidate organisms and their associated reference genomes are given by the GDV and allow estimating the taxonomic identity of the organism.

## Experiments & Results

In this section, we present experimental results that demonstrate on the one hand that MicrobeGPS provides more accurate community composition estimates than previous approaches and on the other hand that MicrobeGPS provides a new quality in analyzing microbial communities. The former is demonstrated on artificial metagenomic data allowing comparison of different tools to a gold standard. For the latter, we reanalyze two different real microbial communities that are challenging and highlight the benefits of MicrobeGPS.

### Comparison on artificial mock community

We compared MicrobeGPS to other methods on the Human Microbiome Project (HMP) Mock Community (MC) metagenomic dataset [21] which is an in vitro synthetic mixture of 21 known bacteria. Although the complexity is below that of typical metagenomes, this dataset is particularly suitable as it includes all typical problems occurring in real data that can hardly be reproduced in simulated datasets such as GC bias, read noise, or mutations. Originally, two mixtures were created: one with even abundance profile, i.e. all organisms are about equally abundant, and one with staggered abundance profile, where the abundances spread over several orders of magnitude. We used the staggered data set because it resembles a natural abundance distribution and is more challenging for the tools. The Illumina sequencing data of the staggered abundance distribution mixture are available from NCBI SRA (accession SRX055381) and contain 7.9 million 75 bp reads. We analyzed the community composition of MC with the state-of-the-art methods Pathoscope [5], MetaPhlAn [4], and mOTUs [10] and compared results with MicrobeGPS. Both MicrobeGPS and Pathoscope build upon the alignment of the metagenomic reads to a database of microbial reference genomes. As reference genome database, we used the HMP [22] and the NCBI [23] bacterial reference genomes for both tools. The reads were mapped with Bowtie 2 (v. 2.1.0, parameters—fast -p 12—no-unal -k 60) to both reference databases for further processing with Pathoscope 1.0 and MicrobeGPS 1.0.0 (Linux binaries). We used default parameters for Pathoscope and configured MicrobeGPS to discard reads mapping to more than 50 references and consider only references with 10 or more unique reads. MetaPhlAn 1.7.7 and mOTUs (standalone version) are shipped with their own curated sets of marker genes for identification. Both tools were configured as suggested in the manuals; MetaPhlAn was set to report species level abundance estimates. On the same hardware, the run time of the marker gene based methods MetaPhlAn (2:19 min) and mOTUs (10:53 min) was lower than for Pathoscope (15:08 min) and MicrobeGPS (31:37 min).

MicrobeGPS reported 24 (HMP) and 23 (NCBI) candidate organisms, Pathoscope reported abundance estimates for 828 (NCBI) and 690 (HMP) reference sequences. MetaPhlAn reported abundance estimates for 32 species, mOTUs for 16 species. We sorted the results by estimated abundances and selected the *N* most abundant identifications of each tool and compared the results to the ground truth. For each *N*, we calculated the sensitivity, false positive rate and precision for the selected set. Plotting the sensitivity against the false positive rate allowed to determine receiver operator characteristic (ROC) for each tool. Additionally, we calculated the F-measure [24]—the harmonic mean of sensitivity and precision—for all *N* and report its maximum for each setup. The quantitative measures are summarized in Table 1.

**Table 1:** Comparison of taxonomic profiling methods on HMP mock community data. We compared the four tools MetaPhlAn, mOTUs, Pathoscope, and MicrobeGPS. For Pathoscope and MicrobeGPS, we used two different reference genome sets, HMP and NCBI (see text). For each tool, we report the overall precision and sensitivity with respect to the ground truth. The maximum F-measure and the area under the ROC curve (AUC) were calculated comparing the *N* most abundant identifications to the ground truth. Highest scores are written in bold font. For Pathoscope, we manually applied a relative abundance cutoff of 10^−5^.

**Method**	**Precision**	**Recall**	**F-measure**	**AUC**
**MetaPhlAn**	0.9048	0.6129	0.8372	0.8738
**mOTUs**	**1.0000**	0.7619	0.8649	0.7619
**Pathoscope (NCBI)**	0.3333	**0.9524**	0.5714	0.8419
**Pathoscope (HMP)**	0.3947	0.7143	0.7222	0.7031
**MicrobeGPS (NCBI)**	0.8261	0.9048	**0.9268**	**0.9032**
**MicrobeGPS (HMP)**	0.7500	0.8571	0.8372	0.8503

**Fig 3:**
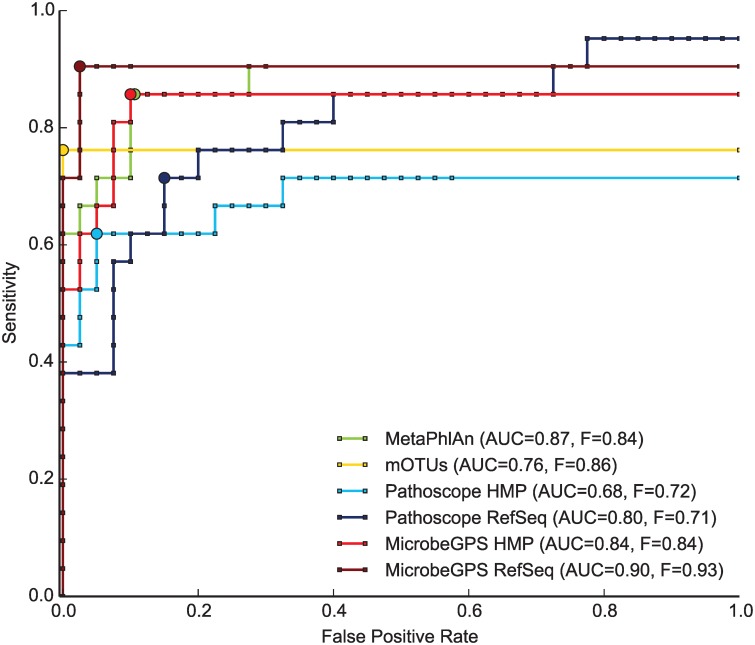
Evaluation of taxonomic profiling tools on *in vitro* metagenomes. We compared MicrobeGPS to Pathoscope [5], MetaPhlAn [4], and mOTUs [10] on the in vitro HMP mock community dataset [21] with known composition. The number of true and false positive identifications among the top *N*, *N* ≤ 60, reported organisms were counted and ROC curves were calculated. The large circle indicates the point of maximum F-measure for each method. MicrobeGPS outperforms the other methods both in terms of AUC and F-measure. MicrobeGPS in combination with the NCBI reference genomes provide the best results, both in terms of F-measure and AUC.

Pathoscope reports substantially longer lists of identified (and sometimes highly similar) reference genomes than the other tools. Often, it is not clear to the user if they are all in the sample or only some of them. To make the results of Pathoscope comparable, we only used the top 60 most abundant organisms for the comparison, which corresponds to a relative abundance cutoff of 10^−5^. For both NCBI and HMP references, MicrobeGPS reports better results than Pathoscope, both in terms of F-measure and AUC (see Fig. 3). Best performance is achieved with NCBI genomes, where MicrobeGPS correctly identified 19 among the first 20 reported organisms. MetaPhlAn is comparable to MicrobeGPS with HMP reference genomes and better than Pathoscope. Although mOTUs had no false positive identification, only 16 out of 21 organisms were found. Even more pronounced than in MetaPhlAn, mOTUs misses the low abundant organisms in the sample that are correctly captured by MicrobeGPS and Pathoscope. This had negative influence on the sensitivity of mOTUs, yielding a comparably low F-measure and AUC.

The main error sources of MicrobeGPS are missing genomes in the reference databases and false positive identifications. For example, HMP lacks *Streptococcus pneumoniae* and *Deinococcus radiodurans* reference genomes. For *D. radiodurans*, MicrobeGPS and Pathoscope report the closest available relative, *Deinococcus deserti*. However, from the Pathoscope output it does not become clear that *D. deserti* is not a perfect match. In contrast, MicrobeGPS reports a low GDV score (0.02) and high mapping error rate (0.12) here, indicating that the candidate is distantly related but not identical to *D. deserti*. The number of false positive identifications from MicrobeGPS is lower than from Pathoscope or MetaPhlAn. On the other hand, MicrobeGPS identifies more correct organisms (NCBI: 19, HMP: 18) in the sample than mOTUs (16). Additionally, the false identifications receive low quality scores in MicrobeGPS and can therefore be spotted easily in the graphical user interface. However, we observed one exception, *Stenotrophomonas maltophilia*, which was detected by all three tools and received a considerably high (0.76) GDV score in MicrobeGPS, but was not listed in the MC dataset ground truth. The unique read matches to *S. maltophilia* were verfied via BLAST search. Therefore, we speculate that *S. maltophilia* was actually present in the sample, probably as contamination.

While other *Streptococci* were present in the dataset and reported by the tools, the very low abundant *S. pneumoniae* was neither detected nor explained by a closely related genome by any of the tools. Instead, MicrobeGPS assigned the *S. pneumoniae* genome to the *Streptococcus mutans* candidate such that it could only be identified as separate organism via manual inspection of the reference genomes supporting the *S. mutans* candidate. Although MetaPhlAn should in principle be able to recover such situations, it fails to make the score and only reports one out of three *Streptococci*.

Taken together, this experiment showed that MicrobeGPS is able to estimate microbial community compositions more accurately than previous approaches. In cases where the identified organism did not agree with the reference genomes, MicrobeGPS quantified the divergence between sequenced organism and reference genome with the GDV score. MicrobeGPS therefore allows differentiating between cases where the suitable reference genome was available and cases where a distantly related genome had to be selected as representative. This differentiation is not possible for MetaPhlAn and Pathoscope.

### Application to human gut microbiome

We further analyzed three human gut metagenomes with MicrobeGPS to evaluate its potential on real data. The datasets (IDs 1122, 2535, and 2638) were acquired from diarrhea patients during the Shiga-toxigenic *E. coli* (STEC) outbreak in Germany, 2011 [25] and were downloaded from NCBI SRA (accessions ERX237457, ERX234998, ERX237461). The datasets contained between 332,257 and 879,176 paired-end reads with length 2×151 bp. Clinical tests identified a *Clostridium difficile* infection in dataset 1122 and high abundances of STEC in datasets 2535 and 2638. We reanalyzed the three datasets and used the NCBI bacterial genomes as reference database. For testing purposes, we also removed the STEC reference sequence from the set of reference genomes. The reads were mapped with Bowtie 2 using the same settings as in the first experiment. MicrobeGPS was configured to report genomes with at least one uniquely matching read.

MicrobeGPS presented 41 candidate organisms for dataset 1122, indicating a higher complexity than the MC dataset. In accordance with the diagnosed *C. difficile* infection, we found a candidate supported by five *C. difficile* genomes and being closest related to *C. difficile* Bl9. However, the reported GDV score is low (0.23) and suggests that the infecting strain differs from all strains available in the database. Higher GDV scores are reported for candidates supported by *Alistipes finegoldii* (0.81) or *Bacteroides vulgatus* (0.79). The most abundant candidate (*A. finegoldii*, 232,090 reads) and the smallest highly relevant candidate (*Eggerthella lenta*, 80 reads) differ by a factor of 2901 in their abundance. Nevertheless, the typical gut bacterium *E. lenta* is a highly valid candidate since most reads mapped uniquely and the reads are homogeneously distributed over the genome (Kolmogorov-Smirnov test p-value below 0.05).

**Fig 4:**
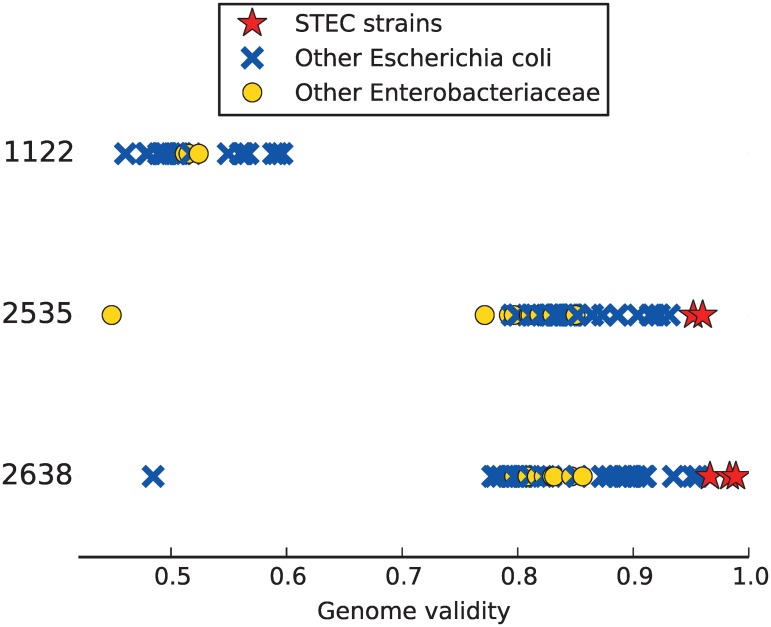
Identification of STEC strains in human gut metagenomes. Three human gut metagenome datasets [25], one of them without (1122) and two with STEC infection (2535, 2638), were analyzed with MicrobeGPS. MicrobeGPS identified one *E. coli*–related candidate in each dataset. In the datasets with STEC infection, MicrobeGPS finds the highest GDV scores for different STEC strains, showing almost perfect agreement between the sequenced organism and reference genome. In dataset 1122, MicrobeGPS only finds other *E. coli* strains with much lower GDV scores, indicating correctly that this sample was not infected with STEC.

The most abundant candidate in datasets 2535 and 2638 was closely related to *E. coli*, however, none of the supporting references could be identified as perfect match since STEC was not part of the reference database. When added to the database, STEC was the most abundant supporting genome and was identified as almost perfect match (GDV = 0.99), as shown in Fig. 4. This is contrasting to dataset 1122, where the *E. coli* candidate had lower abundance and the supporting reference genomes had lower GDV; the STEC genomes were not reported as supporting genomes for the *E. coli* candidate. Since *E. coli* is a common human gut bacterium, we expect that the non-pathogenic *E. coli* was also present in the datasets 2535 and 2638, but was assigned to the same candidate as the high abundant STEC due to the high sequence similarity. This challenging scenario could benefit follow-up analyses with tools specialized to differentiating highly similar organisms in metagenomic samples, such as GASiC [26]. Although no *C. difficile* infection was diagnosed for both samples, MicrobeGPS identified candidates mainly supported by *C. difficile* in both datasets, similarly to dataset 1122. This may not necessarily be wrong since *C. difficile* is a common gut bacterium, but shows that using this set of genomes does not allow the distinction between pathogenic and non-pathogenic *C. difficile* strains.

Altogether, this experiment demonstrated the ability of MicrobeGPS handling real data and identifying the correct strain if available and locating the candidate as good as possible otherwise.

### Reanalysis of Lake Lanier metagenome

Our method is not restricted to the analysis of human-associated microbiomes and is particularly suitable for the exploration of communities with few known reference genomes. Therefore, we reanalyzed the Lake Lanier freshwater metagenome datasets (AUG1, AUG2, SEPT, NOV) [27, 28], a series of four datasets from the same location at different time points. In the original study, the authors assessed the community composition by means of 16S rRNA gene amplicon sequencing and by assembling the metagenomic sequencing reads into contigs and subsequently identifying genes in the sequences. These genes were then searched in databases of all sequenced bacterial and archaeal genomes. Here, MicrobeGPS offers a third way, since the metagenomic sequencing reads are used directly to infer the composition of the community. Therefore, we downloaded the published datasets from NCBI SRA (accessions SRX039150, SRX039152, SRX039381, SRX039382). The dataset sizes were between 13.6 million (SEPT) and 17.1 million (NOV) 2×101 bp paired-end reads. The NCBI bacterial genomes served as reference database for MicrobeGPS and reads were mapped with Bowtie 2 using the same settings as in the previous experiments. MicrobeGPS was used with default settings.

These datasets posed a particularly challenging problem to MicrobeGPS since only about 1% of the reads in each dataset could be mapped to the NCBI bacterial genomes database, indicating that the freshwater metagenome is still far less studied than other environments such as the human microbiome.

**Fig 5:**
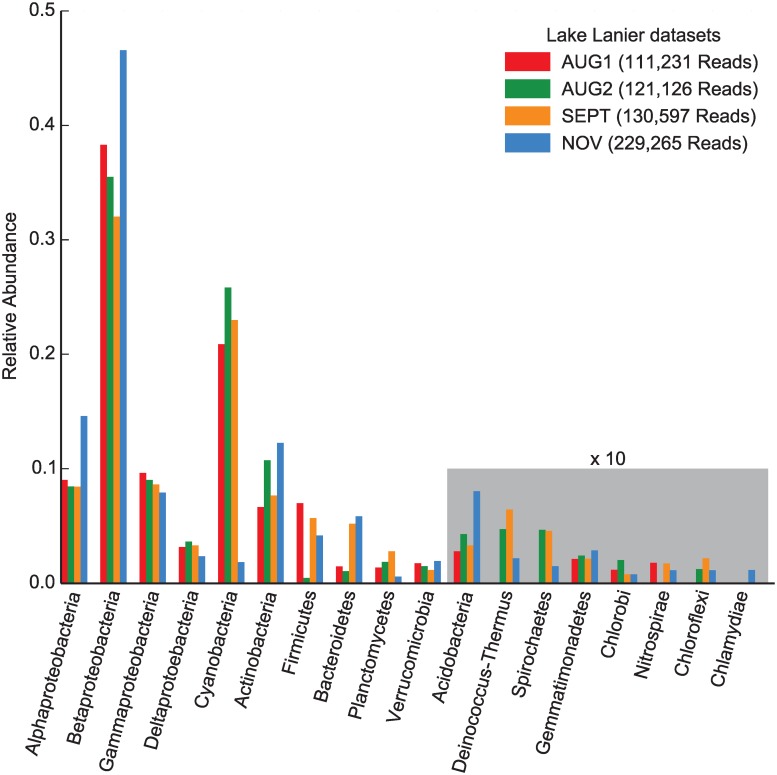
High level taxonomic analysis of freshwater metagenome. We estimated the composition of the four Lake Lanier metagenomic time series datasets [27] on the phylum level (*Proteobacteria* were expanded to the class level), since species accurate identification was not possible due to low coverage and insufficient similarity to reference genomes. MicrobeGPS detected all phyla reported in the original study based on 16S amplicons and assembled contigs with comparable abundances and showed temporal abundance shifts similar to the other approaches.

MicrobeGPS identified between 165 and 238 candidate organisms per dataset from 15 bacterial phyla (see Fig. 5), indicating a much higher community complexity than the MC or STEC datasets. However, the MicrobeGPS quality measures clearly pointed out that the majority of detected candidates diverge strongly from the genomic material in the sample: The highest observed GDV score was 0.45 (*Anabaena* Sp. 90 in AUG1), but the majority of GDV scores were below 0.05. This indicates that the available reference genome sequences are not suitable for species accurate identification in these datasets and the low scores warn the user that each individual candidate should be treated with caution.

Nevertheless, when looking at a more coarse level, we obtained more meaningful results and our observations largely agreed with the results presented in the original studies. Both approaches reported *Proteobacteria* as the most abundant phylum and all originally reported phyla were also identified by MicrobeGPS. Our overall estimated abundances show patterns similar to the assembled contigs approach presented in File S1, Figure S4 in [28]. Also the temporal variations could be reproduced with MicrobeGPS: while the relative abundances of *Proteobacteria*, *Actinobacteria* and *Verrucomicrobia* were relatively stable over all datasets, we also observed a significant drop of abundance for *Cyanobacteria* in the NOV dataset and an increase of *Bacteroidetes. Planctomycetes*, which were hardly detected in the 16S analysis, show highest abundances in the SEPT dataset in both the assembly based and MicrobeGPS analysis.

However, we also observed that the structure of the reference genome database influenced the relative abundance quantification of the different phyla. The 16S approach detected higher fractions of *Cyanobacteria* and *Verrucomicrobia* than MicrobeGPS, but lower fractions of *Proteobacteria*. We attribute this skew to the uneven representation of the phyla in the reference database: the database contained 1104 *Proteobacteria* genomes, but only 71 *Cyanobacteria* and 4 *Verrucomicrobia* genomes. This limitation is inherent to reference genome based approaches and was also observed in the assembly based analysis [28].

For comparison, we profiled the four Lake Lanier datasets with MetaPhlAn and compared the results to the analysis in the original study and to MicrobeGPS. We used the reference sequence database provided by MetaPhlAn in combination with the Bowtie 2 mapper, as suggested in the MetaPhlAn manual. MetaPhlAn was configured to report abundances for all taxonomic levels (—tax_lev = a). All datasets were analyzed separately. The estimated abundances are shown in Table 2.

**Table 2:** Reanalysis of the Lake Lanier metagenomic datasets with MetaPhlAn. The numbers are the estimated percentage abundances on the phylum level. A dash indicates that the phylum was not detected by MetaPhlAn in the dataset.

**Dataset**	**AUG1**	**AUG2**	**SEPT**	**NOV**
**Cyanobacteria**	73.3	59.9	59.3	3.6
**Proteobacteria**	14.2	19.9	30.7	74.8
**Bacteroidetes**	9.5	10.1	10	14.9
**Chlamydiae**	3	-	-	1.7
**Actinobacteria**	-	7.1	-	5
**Chloroflexi**	-	3	-	-

In contrast to MicrobeGPS, MetaPhlAn detected only 3–5 phyla and 6–9 different species with varying abundances in the datasets without providing any information about the accuracy of the results. These results suggest a community complexity far lower than what one would expect for a freshwater metagenome and than what was observed in the original study and the MicrobeGPS analysis. Furthermore, most MetaPhlAn abundance estimates do not coincide with the other approaches. For example, *Cyanobacteria* abundance was estimated between 59% and 74% in the first three datasets, three times higher than MicrobeGPS. *Actinobacteria* were only detected in AUG2 and NOV and *Verrucomicrobia* were not found at all, although the two other approaches reported considerable abundances for both phyla in all datasets.

This experiment showed that MicrobeGPS estimates microbial community compositions similarly to traditional approaches like manual assembly and 16S based taxonomic profiling even on challenging datasets, where other methods have severe problems.

## Discussion

Reference based taxonomic profiling is currently the most accurate way of assessing the composition of microbial communities as it allows—in principle—strain accurate identification of organisms in metagenomes. However, current tools tend to overestimate their accuracy as they report a specific strain or species as present when the dataset contains a previously unknown related organism. We demonstrated that the user has in practice no possibility to differentiate between cases with strain accurate identification and identification of a related organism.

Therefore, we introduced MicrobeGPS, a novel approach to the taxonomic profiling problem which is beneficial in two ways. First, MicrobeGPS provides more accurate community composition estimates than other reference based methods. Second and more important, MicrobeGPS identifies candidate organisms by their sequencing depth and recruits known reference genomes supporting the candidate. The quality measures calculated for each detected candidate organism allow the user to judge the quality and reliability of the identification. Thereby, unknown organisms that are not represented in current reference genome collections can be evaluated critically and their supporting reference genomes provide valuable information about the organism’s taxonomic affiliation. Here, MicrobeGPS is far ahead of related tools that only report estimated abundances for hundreds of genomes without any other quality information, and thus contributes to the trustworthiness of already powerful reference-based metagenomic analyses.

The GDV score turned out as a valuable measure of sequence disagreement, quantifying similarity between the organism in the sample and the available reference genome. Compared to the average nucleotide identity (ANI, see [29]), a common measure for genomic similarity which only operates on the regions shared between genomes, the GDV score is designed to compare an existing genome to a set of sequencing reads and is particularly applicable when the genome shares only small regions with the sequenced organism. If the only similarity between the sequenced organism and the reference genomes is a single gene transferred with only very few mutations from one of the species available as reference to the organism in the sample, the ANI assigns a high identity as it only considers regions shared between the genomes. In contrast, the GDV is close to zero since only a very small part of the whole reference genome is actually present in the dataset.

Our experiments demonstrated that MicrobeGPS provides more concise and accurate results than previous approaches on *in vitro* microbial communities with known composition. On real datasets, MicrobeGPS is able to provide strain accurate identifications for well-studied species (such as *E. coli*) with sufficient reference genomes and at the same time coarse identification of organisms where no perfectly matching reference genome is available. This dynamic adaptation to the structure of the reference genome set is comparable to lowest common ancestor approaches while being more informative about the quality and reliability of each candidate. When no accurate identification on low taxonomic levels is possible, as in the Lake Lanier experiment, the user can analyze the dataset on a higher level (e.g. *phylum*). Here, MicrobeGPS produces community composition estimates that are comparable to established approaches involving assembly of the sequence reads into contigs or the analysis of 16S rRNA amplicon data.

The benefits of MicrobeGPS come at the cost of an increased run time and memory footprint: on the same hardware Pathoscope is about 50% faster than MicrobeGPS. Approximately one third of the run time is used to map the reads, one third to transform the SAM file into the datastructures required by MicrobeGPS, and the last third to calculate the GDV scores and the clustering. The interactive graphical user interface requires that all mapping information is kept in memory. The memory consumption strongly depends on the number of shared read matches; in our experiments we observed peak memory consumptions between 1 GB (Lake Lanier datasets) and 38 GB (MC dataset). Thus, smaller datasets can be processed on a laptop computer while larger datasets may require a larger (e.g. workstation) computer.

In particular in comparison to other taxonomic profiling methods, such as MetaPhlAn, Kraken, or Taxy [30], it would be desirable for MicrobeGPS to have lower runtime. However, the advantage of MicrobeGPS over other methods originates from taking full advantage of the whole reference genome sequence. Therefore, a strategy as used in MetaPhlAn would not be applicable. Yet, a k-mer based approach, as used in Kraken or Taxy, might also be applicable for MicrobeGPS. Instead of searching for core reads (CR), one could search for core words, i.e. k-mers found in different metagenomes with similar coverage. In our opinion, such an approach could have the potential to speed up MicrobeGPS while possibly keeping the strength of the current, alignment-based method.

Despite the significant improvement on the status quo, MicrobeGPS still suffers from a problem that is common to all reference based taxonomic profiling approaches—the influence of the reference database. Especially in the two real data experiments, we saw that MicrobeGPS is able to identify on species or strain level when the database contains sufficient reference genomes, but only finds distantly related organisms if no suitable reference genome is available. Furthermore, unbalanced databases skew the abundance estimates, where taxonomic groups with many reference genomes appear more abundant than groups with few reference genomes. However, MicrobeGPS reduced the influence of the reference genome database in comparison to recent methods and makes the problem of missing reference genomes tractable.

While reference based taxonomic profiling could previously only be applied to microbial communities with known structure where the reference genomes of the most dominant organisms are known, our experiments demonstrated that MicrobeGPS can also be used for taxonomic profiling of less explored communities such as the Lake Lanier metagenome. This result in combination with the growing databases of reference genomes could pave the way for the application of reference based taxonomic profiling beyond applications such as clinical diagnostics [5]. Compared to traditional methods such as 16S rRNA based profiling or lowest common ancestor approaches, we showed that our method has the advantage that strain specific identification is in principle possible, but can fall back to more coarse identifications when reference genomes are missing. Yet, the approach may still not be applicable to microbial communities with extremely complex composition and only few known genomes, such as soil metagenomes.

## Supporting Information

S1 Text Sequencing depth estimation for low abundant organisms. The framework introduced in [12] allows estimating the local sequencing depth and GDV score of a reference genome down to 0.2× sequencing depth. Here, we present an extension of the framework such that sequencing depths and GDV scores of genomes with down to 0.01× sequencing depth can be estimated.
(PDF)

## References

[1] Mason OU, Hazen TC, Borglin S, Chain PS, Dubinsky EA, et al (2012) Metagenome, metatranscriptome and single-cell sequencing reveal microbial response to Deepwater Horizon oil spill. ISME J 6: 1715–1727. 10.1038/ismej.2012.59 22717885 PMC3498917

[2] Dodsworth JA, Blainey PC, Murugapiran SK, Swingley WD, Ross CA, et al (2013) Single-cell and metagenomic analyses indicate a fermentative and saccharolytic lifestyle for members of the OP9 lineage. Nat Commun 4: 1854 10.1038/ncomms2884 23673639 PMC3878185

[3] Nagarajan N, Pop M (2013) Sequence assembly demystified. Nature Reviews Genetics 14: 157–167. 10.1038/nrg3367 23358380

[4] Segata N, Waldron L, Ballarini A, Narasimhan V, Jousson O, et al (2012) Metagenomic microbial community profiling using unique clade-specific marker genes. Nat Methods 9: 811–814. 10.1038/nmeth.2066 22688413 PMC3443552

[5] Francis OE, Bendall M, Manimaran S, Hong C, Clement NL, et al (2013) Pathoscope: species identification and strain attribution with unassembled sequencing data. Genome Res 23: 1721–1729. 10.1101/gr.150151.112 23843222 PMC3787268

[6] Bonfert T, Csaba G, Zimmer R, Friedel CC (2013) Mining RNA–seq data for infections and contaminations. PLOS ONE 8: e73071 10.1371/journal.pone.0073071 24019895 PMC3760913

[7] Wood D, Salzberg S (2014) Kraken: ultrafast metagenomic sequence classification using exact alignments. Genome Biol 15: R46 10.1186/gb-2014-15-3-r46 24580807 PMC4053813

[8] Von Mering C, Hugenholtz P, Raes J, Tringe S, Doerks T, et al (2007) Quantitative phylogenetic assessment of microbial communities in diverse environments. Science 315: 1126–1130. 10.1126/science.1133420 17272687

[9] Simon C, Daniel R (2011) Metagenomic analyses: past and future trends. Appl Environ Microbiol 77: 1153–1161. 10.1128/AEM.02345-10 21169428 PMC3067235

[10] Sunagawa S, Mende DR, Zeller G, Izquierdo-Carrasco F, Berger SA, et al (2013) Metagenomic species profiling using universal phylogenetic marker genes. Nat Methods 10: 1196–1199. 10.1038/nmeth.2693 24141494

[11] Wu YW, Ye Y (2011) A novel abundance-based algorithm for binning metagenomic sequences using l-tuples. J Comp Biol 18: 523–534. 10.1089/cmb.2010.0245 PMC3123841 21385052

[12] Lindner MS, Kollock M, Zickmann F, Renard BY (2013) Analyzing genome coverage profiles with applications to quality control in metagenomics. Bioinformatics 29: 1260–1267. 10.1093/bioinformatics/btt147 23589648

[13] Huson DH, Auch AF, Qi J, Schuster SC (2007) MEGAN analysis of metagenomic data. Genome Res 17: 377–386. 10.1101/gr.5969107 17255551 PMC1800929

[14] Metzker ML (2009) Sequencing technologies–the next generation. Nat Rev Genet 11: 31–46. 10.1038/nrg2626 19997069

[15] Langmead B, Salzberg SL (2012) Fast gapped-read alignment with Bowtie 2. Nat Methods 9: 357–359. 10.1038/nmeth.1923 22388286 PMC3322381

[16] Sedlazeck FJ, Rescheneder P, von Haeseler A (2013) NextGenMap: fast and accurate read mapping in highly polymorphic genomes. Bioinformatics 29: 2790–2791. 10.1093/bioinformatics/btt468 23975764

[17] Siragusa E, Weese D, Reinert K (2013) Fast and accurate read mapping with approximate seeds and multiple backtracking. Nucleic Acids Res 41: e78–e78. 10.1093/nar/gkt005 23358824 PMC3627565

[18] Lambert D (1992) Zero-inflated Poisson regression, with an application to defects in manufacturing. Technometrics 34: 1–14. 10.2307/1269547

[19] MacQueen J (1967) Some methods for classification and analysis of multivariate observations. In: Proceedings of the fifth Berkeley symposium on mathematical statistics and probability. California, USA, volume 1, pp. 281–297.

[20] Johnson SC (1967) Hierarchical clustering schemes. Psychometrika 32: 241–254. 10.1007/BF02289588 5234703

[21] Methé BA, Nelson KE, Pop M, Creasy HH, Giglio MG, et al (2012) A framework for human microbiome research. Nature 486: 215–221. 10.1038/nature11209 22699610 PMC3377744

[22] Nelson KE, Weinstock GM, Highlander SK, Worley KC, Creasy HH, et al (2010) A catalog of reference genomes from the human microbiome. Science 328: 994–999. 10.1126/science.1183605 20489017 PMC2940224

[23] Pruitt KD, Tatusova T, Brown GR, Maglott DR (2012) NCBI Reference Sequences (RefSeq): current status, new features and genome annotation policy. Nucleic Acids Res 40: D130–D135. 10.1093/nar/gkr1079 22121212 PMC3245008

[24] Van Rijsbergen CJ (1979) Information Retrieval. London: Butterworths, 2nd edition.

[25] Loman NJ, Constantinidou C, Christner M, Rohde H, Chan JZM, et al (2013) A culture-independent sequence-based metagenomics approach to the investigation of an outbreak of shiga-toxigenic Escherichia coli O104:H4. JAMA 309: 1502–1510. 10.1001/jama.2013.3231 23571589

[26] Lindner MS, Renard BY (2013) Metagenomic abundance estimation and diagnostic testing on species level. Nucleic Acids Res 41: e10 10.1093/nar/gks803 22941661 PMC3592424

[27] Oh S, Caro-Quintero A, Tsementzi D, DeLeon-Rodriguez N, Luo C, et al (2011) Metagenomic insights into the evolution, function, and complexity of the planktonic microbial community of Lake Lanier, a temperate freshwater ecosystem. Appl Environ Microbiol 77: 6000–6011. 10.1128/AEM.00107-11 21764968 PMC3165412

[28] Poretsky R, Rodriguez-R LM, Luo C, Tsementzi D, Konstantinidis KT (2014) Strengths and limitations of 16S rRNA gene amplicon sequencing in revealing temporal microbial community dynamics. PLOS ONE 9: e93827 10.1371/journal.pone.0093827 24714158 PMC3979728

[29] Konstantinidis KT, Tiedje JM (2005) Genomic insights that advance the species definition for prokaryotes. Proc Natl Acad Sci USA 102: 2567–2572. 10.1073/pnas.0409727102 15701695 PMC549018

[30] Meinicke P, Aßhauer KP, Lingner T (2011) Mixture models for analysis of the taxonomic composition of metagenomes. Bioinformatics 27: 1618–1624. 10.1093/bioinformatics/btr266 21546400 PMC3106201

