# Supplementary material for: Metagenomic Profiling of Known and Unknown Microbes with MicrobeGPS
Source: PLoS One. 2015 Feb 2;10(2):e0117711. doi: 10.1371/journal.pone.0117711 (PMC4314203; doi:10.1371/journal.pone.0117711)
Supplement: S1 Text — The framework introduced in [12] allows estimating the local sequencing depth and GDV score of a reference genome down to 0.2× sequencing depth. Here, we present an extension of the framework such that sequencing depths and GDV scores of genomes with down to 0.01× sequencing depth can be estimated. (PDF) [file pone.0117711.s001.pdf]

Supplementary Text

Reference-based characterization of known and unknown microbes in metagenomes with MicrobeGPS

Martin S. Lindner & Bernhard Y. Renard*

1 Sequencing depth estimation for low abundant organisms

In our previous work, [1], we presented an analyze the observed coverage depth on a reference genome by fitting mixtures of discrete probability distributions to the genome coverage profile (GCP), the histogram of coverage depths on the genome. As stated there, calculating the GCP, i.e. transforming the observed position wise coverage depth values into the histogram of observed coverage depth values, summarizes the information hidden in the coverage depth of the genome. The conducted experiments [1] suggested that this condensed view brings benefit for the analysis of sequencing data and that interesting features such as the genome–dataset validity can be derived from GCPs. However, this transformation comes with a loss of information. Before, we had one coverage depth value for each position in the genome, which is some million datapoints for a bacterial genome. In contrast, the number of datapoints in the GCP is the highest observed coverage depth on the genome. Even for ultra-deep sequencing, the number of GCP datapoints will not exceed several thousands. In the more common case of lower sequencing depths, the number of GCP datapoints becomes very critical, since all parameters in the mixture distributions (Equation 1 in [1]) must be estimated from these datapoints. For example, the distribution consisting of zero, Poisson, and negative binomial with tail (`zpnt`) has 6 parameters and therefore requires that a sufficient number of positions with coverage depth $6\times$ is observed in the dataset. In order to compensate for noisy data, higher sequencing depths are desirable. In the extreme case when the sequencing depth is so low that the reads do not overlap, the only possibility is to fit a zero-inflated Poisson (`zp`) model since the GCP contains only the datapoints 0 and 1, which allows determining solutions for two parameters. These parameters would be very error prone in practice due to noise.

*Contact: `RenardB@rki.de`

In the following sections we develop a model similar to the GCP case, that allows to estimate the sequencing depth and the genome–dataset validity score [1] as used in the main text. This model is designed for extremely low coverage depths and should therefore complement the existing model. The mathematical procedures, i.e. the modified EM algorithm, are identical; we only use different data to construct the histogram and fit different distributions to that histogram.

1.1 A new model for low coverage depths

Estimating the genome–dataset validity (GDV) with the previously described approach can be problematic for very low coverage depths: here, the approach with calculating the GCP and fitting, e.g., a zero-inflated negative binomial distribution with tail (`znt`) model, is not appropriate since we do not expect to have sufficient datapoints for robust parameter estimation. To increase the number of datapoints, we can look at the starting positions of the reads mapped to the genome, as shown in Figure 1 (a).

Under the assumption that the sequencing depth is homogeneous over the entire genome, the probability that a mapped read starts at a certain position on the genome is equal for all positions. We denote this read start probability as p. Let us consider a read starting at position $X = 0$. The probability that the next read starts at position $X = k$ follows a geometric distribution with parameter p. This becomes clear when considering that if the *next* read starts at position $X = k$, no read starts at positions $X = 0..k-1$. Therefore, the distances between the starting positions of mapped reads are geometrically distributed.

In analogy to the GCP, we call the histogram of distances between read starting positions the Read Distance Profile (RDP). When reads of an organism are mapped to its reference genome, we can simply estimate the parameter p of the geometric distribution by making use of the formula for the expected value of the geometric distribution: $\bar{D} = E(X) = \frac{1-p}{p}$. However, if we expect more than one contribution to the RDP (e.g., reads from two different organisms map to the same genome), we have to set up a mixed model similar to Equation 1 in [1], consisting of two or more geometric distributions. In fact, our framework presented there can be adapted to fit RDPs simply by using models consisting solely of geometric distributions. Parameter estimation is identical to the GCP case.

Since the number of reads mapped to a genome is typically in the order of hundreds or thousands even in cases of ultra-low coverage depths and the distances are very large, we have increased the number of datapoints for parameter estimation significantly with this approach. We will show in the experiments that this approach allows can be applied at much lower coverage depths than the GCP approach.

1.2 Genome–dataset validity from RDPs

The GDV score introduced in [1] is a useful tool for estimating the similarity between a set of reads and a reference genome. However, since the estimation involves fitting complex models to GCPs, we expect decreasing accuracy for lower coverage depths. Here

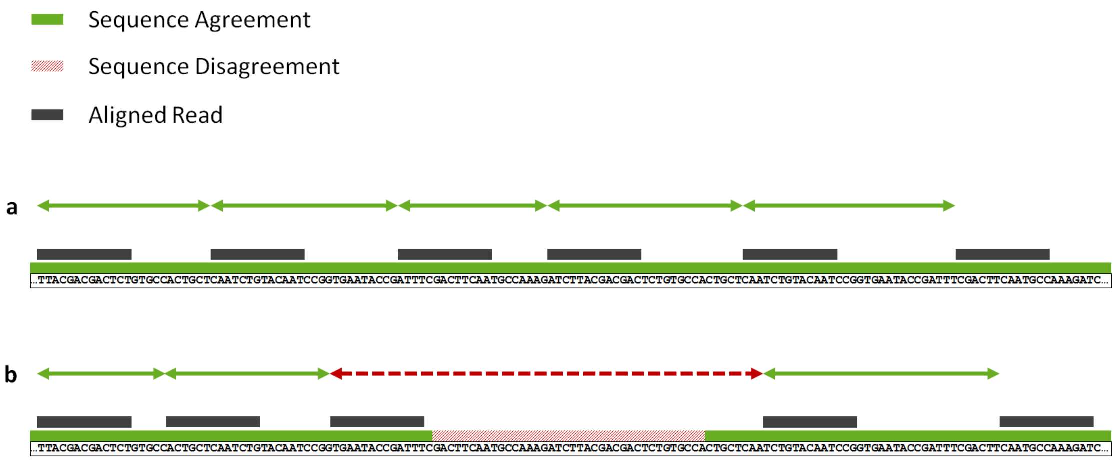

Figure 1. Distances between read start positions. (a) An organism is sequenced with low and homogeneous sequencing depth and its reference genome is available. Then, the distances between neighboring read start positions in a shotgun sequencing experiment can be described by a geometric distribution. (b) When the reference genome differs from the sequenced organism, there will be islands of sequence agreement (green) divided by gaps of sequence disagreement (red). The distances between neighboring reads on the islands (green arrows) still follow a geometric distribution, disturbed by the distances spanning the gaps (red arrow).

we show that it is also possible to calculate the GDV from RDPs and should therefore be accessible for much lower coverage depths.

Similarly to the role of the zero distribution in GCPs, we have to estimate the fraction of the genome that is not covered by reads. Therefore, we have to quantify the gaps between the parts of the genome that could potentially be covered by reads (see Figure 1 b). Here, we introduce a heuristic and assume that the gap lengths also follow a geometric distribution. This is motivated by the assumption that shorter gaps should be more frequent than longer gaps.

To calculate the GDV, it is sufficient to fit two geometric distributions to the RDP: the first distribution fits the distances between reads lying on a contiguous sequence fragment of the reference (visualized by green arrows in Figure 1 b). The second distribution fits the distances between reads lying on neighboring sequence fragments divided by a gap of foreign sequence (red arrow in Figure 1 b). Let α_1 and p_1 denote the estimated parameters of the geometric distribution fitting the distances between reads on the same fragment and let α_2 and p_2 denote the estimated parameters of the geometric distribution fitting the distances between reads on neighboring fragments. Here, we also make the assumption that the distances between reads on the same fragment are typically lower than the distances between reads on neighboring fragments and therefore $p_1 > p_2$. One way to estimate the GDV is to calculate the expected number of reads mapping to the genome under the assumption that the reference genome contained no foreign sequences and to relate this number to the observed number of reads R that were actually mapped to the genome. The expected number of reads is the genome length L divided by the expected distance between reads: $\bar{D} = \frac{1-p_1}{p_1}$. Therefore, we write the GDV for low coverage depths as follows:

$$\mathrm{GDV}_{lc} = \frac{R \cdot \bar{D}}{L} = \frac{R \cdot (1 - p_1)}{p_1 \cdot L}. \tag{1}$$

The coverage depth on the covered sequence fragments can be calculated using the (average) read length RL:

$$cov_{lc} = \frac{RL \cdot p_1}{1 - p_1}.$$

In practice, we may add a third geometric distribution to the model, which accounts for higher coverage depths, e.g., on highly conserved genes. The third geometric distribution does not influence the formulas to calculate the GDV and the sequencing depth. However, it will fit the short distances between reads and therefore influence the parameter estimation. We test this approach on low sequencing depth data and present the experiments and results in the following sections.

2 Experiment: GDV at low coverage depths

In this experiment, we evaluate how well the GDV score can be calculated for genomes with very low coverage depth using the distance-based approach presented above. In [1], we showed that the GDV score could only be calculated robustly for coverage depths as

low as 0.2×. In principle, the distance-based approach should be able to estimate the GDV for even smaller coverage depths, therefore, we assessed its robustness systematically.

We simulated two random genome sequences from the alphabet {A,C,G,T}: a genome A with fixed length 4,600,000 bp (approximate length of the *E. coli* genome), which serves as reference genome, and a smaller genome B with variable length. The sequence of genome B was cut into 20 fragments that were integrated into genome A. The length of genome B was chosen such that A had a defined GDV for reads sequenced from B. We simulated pairs of genomes A and B with validities $GDV = 0.8,\ 0.6,\ 0.4,\ 0.2,\ 0.1$. For each GDV, we simulated sets of 72 bp reads from genome B, such that the sequencing depth on B was $cov = 5\times,\ 2\times,\ 1\times$, 0.5×, 0.2×, 0.1×, 0.05×, 0.02×, 0.01×, 0.005×, 0.002×, 0.001×.

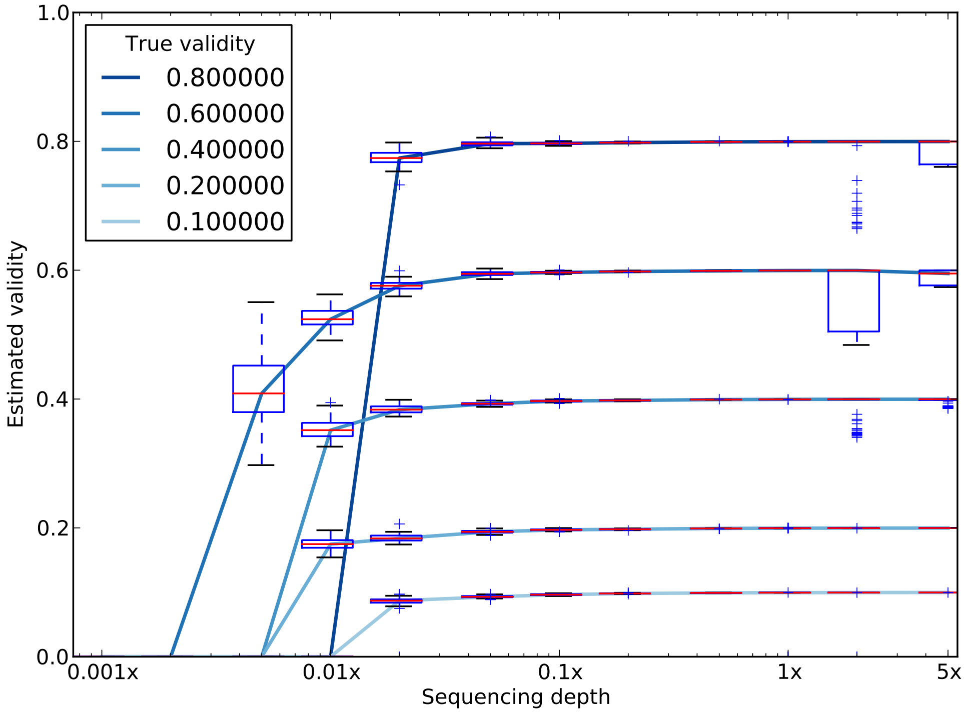

Figure 2. GDV estimation at low coverage depths. We simulated pairs of reference genomes and datasets with fixed coverage depth and reference genomes with defined GDV with respect to the dataset. GDV scores were estimated for each combination using the distance-based approach. The distance-based and true GDV scores agree if the coverage depth exceeds a certain minimum depth. This minimum depth depends on the GDV, where the intermediate GDV scores (0.2 and 0.4) can be robustly estimated at much lower depths than the extreme scores.

For each combination of ground truth and coverage depth, we calculated the GDV and

the estimated coverage depth using the distance-based approach above. The experiment was repeated 100 times. The estimated GDV scores are shown in Figure 2. Here, we see that the quality of the GDV estimate depends both on the coverage depth and on the GDV itself. Intermediate GDV scores (0.2 and 0.4) can be correctly estimated at much lower coverage depths than more extreme validities. For a GDV of 0.2, a minimum of $0.01\times$ coverage depth is required to estimate the GDV with below 10% error in 50% of all cases. In general, a minimum sequencing depth of $0.02\times$ was sufficient to estimate the GDV with less than 10% error.

This result is remarkable when we consider that the coverage-depth-based approach applied in [1] only provided stable estimates down to $0.2\times$ coverage depth. This means that the new approach lowered this limit by up to a factor of 20 compared to calculating the GDV from the coverage depth profile. For a genome with 0.2 GDV and 72 bp reads, this means that only one read every 7,200 bp is required to estimate the GDV correctly.

There are two effects that influence the required minimum coverage depth: For low GDV scores, only small fractions of the genome are covered with reads at all and the covered regions are smaller than for high GDV. At very low depths, it becomes less likely that two or more reads are mapped to one contiguous fragment; at this point, we start to observe mostly distances between reads on different fragments, which makes it impossible to infer the local coverage depth on the covered fragments. On the other hand, for high GDV scores, the gaps between the covered fragments become very small. If the coverage depth is too low, the distances between neighboring reads may become larger than the gaps between covered fragments, our assumption fails and makes it impossible to mathematically distinguish the distribution of gaps between reads on the same fragment from the gaps between reads on different fragments. We presume that our approach of calculating the GDV is close to the technical limit that can be reached with any approach based on read mapping.

References

1. Lindner MS, Kollock M, Zickmann F, Renard BY (2013) Analyzing genome coverage profiles with applications to quality control in metagenomics. Bioinformatics 29: 1260–1267.
